# Evaluation of the feasibility of human papillomavirus sponge‐type self‐sampling device at Japanese colposcopy clinics

**DOI:** 10.1111/jog.15496

**Published:** 2022-12-15

**Authors:** Nobuyoshi Ozawa, Tetsuji Kurokawa, Hitoshi Hareyama, Hiroshi Tanaka, Michihiro Satoh, Hirohito Metoki, Mitsuaki Suzuki

**Affiliations:** ^1^ Ozawa Women's General Clinic Sendai Miyagi Japan; ^2^ Department of Obstetrics and Gynecology, Faculty of Medical Sciences University of Fukui Fukui Fukui Japan; ^3^ Sapporo Women's JR‐Tower Clinic Sapporo Hokkaido Japan; ^4^ Tanaka Women's Clinic Saga Saga Japan; ^5^ Division of Public Health, Hygiene and Epidemiology, Faculty of Medicine Tohoku Medical and Pharmaceutical University Sendai Miyagi Japan; ^6^ Japan Association of Obstetricians and Gynecologists Tokyo Japan

**Keywords:** cervical cancer screening, CIN, HPV testing, human papillomavirus, self‐sampling

## Abstract

**Aim:**

Self‐sampling human papillomavirus (HPV) testing has been introduced for cervical cancer screening worldwide. In Japan, there are two types (brush and sponge) of HPV self‐sampling devices. However, the recommended type for cervical cancer screening remains unclear. This study aimed to evaluate the feasibility of the HPV self‐sampling device–sponge type (HSD‐ST). Additionally, we aimed to examine the positive rate (sensitivity) for cervical intraepithelial neoplasia (CIN) 2 or worse using the HSD‐ST. Finally, we aimed to perform a questionnaire survey regarding the usability of the HSD‐ST.

**Methods:**

We included 165 women who underwent HPV testing at one of three gynecologic clinics. First, the women used the HSD‐ST and completed a questionnaire regarding its usability. Subsequently, they underwent physician‐sampling HPV testing and cytology. We examined the agreement rate of HPV positivity between self‐ and physician‐sampling HPV testing.

**Results:**

The HPV‐positive rates of self‐ and physician‐collected samples were 59.4% and 62.4%, respectively, with an overall concordance rate of 88.5% and a calculated kappa coefficient of 0.76, indicating high concordance. Moreover, the positive (sensitivity) rates for CIN2 or worse were 81.4% and 89.8% in the self‐ and physician‐collected samples, respectively.

**Conclusions:**

Our findings demonstrated the feasibility and usability of the HSD‐ST.

## INTRODUCTION

High‐risk human papillomavirus (HPV) is a major cause of cervical cancer. Moreover, most cervical squamous cell carcinomas (>90%–95%) are related to HPV.^1^ The incidence of cervical cancer in Japan has continuously increased over the last 10 years.^2^ In 2019, the cervical cancer screening rate in Japan was approximately 40%.^3^ Conversely, the cervical cancer screening rates exceed 70% in other developed countries (including the United States, Germany, and France) where HPV testing has been incorporated into health screening processes.^4^ The most common screening methods in these countries include HPV testing alone or combined with conventional cytology. Additionally, to increase the screening rate, measures such as the call and recall system have been used to encourage participation in screening programs. Furthermore, several countries have integrated the self‐sampling of cervical cells for HPV testing into screening programs to reduce psychological and physical barriers regarding testing.

In 2009, the Japanese Guideline for Cervical Cancer Screening recommended cytology alone.^5^ In 2011, the Gynecologic Oncology Committee of the Japanese Society of Obstetrics and Gynecology recommended combining cytology with HPV testing. However, the Japanese Ministry of Health, Labor and Welfare still recommend cytology alone, and approximately 10% of municipalities currently provide a combination of cytology and HPV testing. The Japanese Guideline for Cervical Cancer Screening, based on efficacy assessment, was updated in 2020, and recommends HPV testing alone as a Grade A recommendation.^6^ However, the guidelines stipulate that, in principle, physicians should collect samples given insufficient evidence regarding HPV self‐sampling in Japan. Since self‐sampling involves fewer psychological and physical barriers than physician‐sampling, widespread use of HPV self‐sampling could increase the cervical cancer screening rate in Japan.

There are two types of self‐sampling devices (brush and sponge). However, the recommended type for cervical cancer screening remains unclear. This study aimed to evaluate the feasibility of the HPV self‐sampling device–sponge type (HSD‐ST). Moreover, we aimed to examine the sensitivity of HSD‐ST for CIN2 and perform a questionnaire survey of the usability of the HSD‐ST.

## MATERIALS AND METHODS

### Study design

We included women who visited one of the three participating gynecologic clinics where colposcopy was available; moreover, the study population was suspected of being at a high risk of HPV infection. Women aged 20–50 years were included. We excluded women during menstruation or within 1 week after menstruation, pregnant women, and those who had undergone total hysterectomy. Finally, 165 women were enrolled from May to November 2020. All participants provided written informed consent.

**FIGURE 1:**
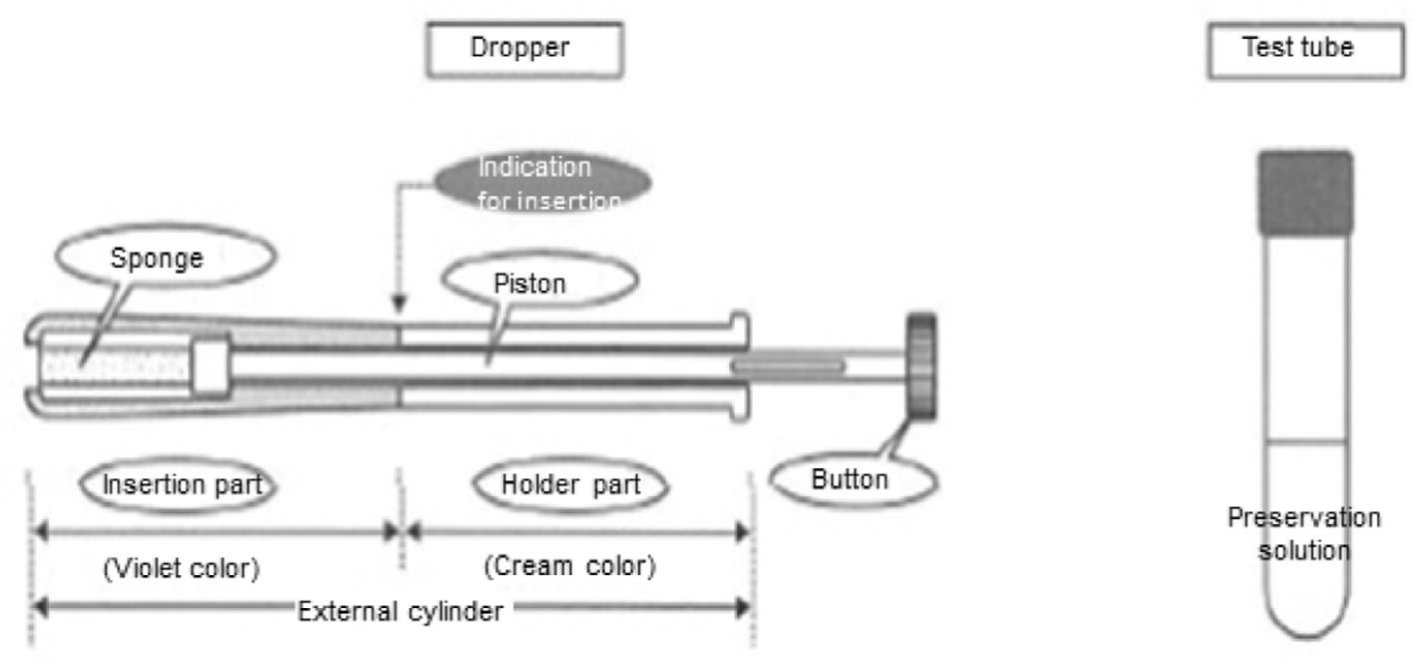
HPV self‐sampling kit using sponge device (HSD‐ST). Dropper: polyethylene, polypropylene. Sponge: natural rubber sponge. HPV, human papillomavirus.

The participants were provided with the HSD‐ST (Home Smear Set Plus®, Asica Medical Industry Co., Ltd.) (Figure 1), the respective instruction manual, the questionnaire. They were asked to perform self‐sampling of cervical cells and completed a questionnaire regarding the usability of the HSD‐ST. Subsequently, physician‐sampling of cervical cells was performed. HPV testing was then conducted using self‐ and physician‐collected samples. Self‐sampling was performed before physician‐sampling because some participants were expected to undergo various procedures, including colposcopy, histological examination, and conization, after examination by the physician.

Cytology and histological results were received within 3 months and 1 year, respectively, before obtaining informed consent. Cytology and histological tests were performed when considered necessary by the physician, if participants were suspected of having cervical cancer on the day informed consent was provided or if participants were suspected of having cervical cancer based on the HPV status after the day of informed consent.

Cytological findings were classified using the Bethesda System and histological findings were classified based on the General Rules for Clinical and Pathological Management of uterine cervical cancer fourth edition.

The study conformed to the ethical guidelines for medical and biological research involving human participants. The study protocol, informed consent sheet, and other ethics‐related documents were approved by the ethics committee of Kitamachi Clinic and the head of each medical institution (Project no. CMI07279).

### Cell sampling devices

Most participants were not accustomed to using self‐sampling devices. Since the type of device may influence the results, we only used the HSD‐ST (Figure 1). The physicians used Cervex Brush® (Becton, Dickinson, and Company) as the sampling device for cervical cell collection. The test tube of the device, in which the self‐collected cervical cells were stored, and the ThinPrep® vial (Hologic, Inc.) in which physician‐collected cervical cells were stored, were refrigerated until HPV testing. They were triple‐packaged and sent to a laboratory for HPV testing within 1 week based on the handling procedures for infectious specimens.^7^

### HPV testing

HPV testing of self‐ and physician‐collected samples was performed using the Cobas 4800 HPV system (Roche Diagnostics KK) at CMIC Pharma Science Co., Ltd.^8^

Further, HPV DNA testing of cervical cells was performed. The levels of high‐risk HPV genotypes, that is, HPV16, HPV18, and other HPV genotypes (HPV31, 33, 35, 39, 45, 51, 52, 56, 58, 59, 66, and 68), were measured.

### Endpoints

The primary endpoint was the overall agreement rate between self‐sampled and physician‐sampled test results, which was calculated as follows: the sum of positive and negative concordant samples/total number of samples. The secondary endpoints were the positive and negative concordance rates calculated using the physician‐sampling method for HPV testing as the benchmark. The positive concordance rate was calculated as the proportion of positive concordant samples to positive physician‐collected samples while the negative concordance rate was calculated as the proportion of negative concordant samples to negative physician‐collected samples. We performed stratified analyses of the self‐ and physician‐sampling test results.

### Statistical analyses

Statistical analyses were performed using SAS Ver.9.4. The overall, positive, and negative concordance rates were evaluated using the kappa coefficient and the confidence interval based on the Clopper–Pearson exact method (*F* distribution). Paired nominal data were tested using McNemar's test or binomial test, as appropriate.

## RESULTS

### Patient characteristics

Table 1 shows the patient characteristics, including age and the results of cytology and pathological classification. The mean age (±standard deviation) of the participants was 35.5 ± 6.8 years (range: 21–50 years). A total of 162 women underwent cytology. The cytological results were as follows: Negative for intraepithelial lesion or malignancy (NILM), 43.8%; atypical squamous cells of undetermined significance (ASC‐US), 9.3%; atypical squamous cells, not excluding a high grade squamous intraepithelial lesion (ASC‐H), 3.1%; low‐grade squamous intraepithelial lesion (LSIL), 16.0%; high‐grade squamous intraepithelial lesion (HSIL), 27.8%; squamous cell carcinoma (SCC), 0%; and others, 0%. Abnormal cytology (ASC‐US or worse) was observed in 91 (56.2%) of the patients.

**TABLE 1:** Patient characteristics

Number of participants	*n* = 165
**Age (years)**	*n* (%)
20–29	36 (21.8)
30–39	84 (50.9)
≥40	45 (27.3)
**Cytology**	162 (100)
NILM	71 (43.8)
ASC‐US	15 (9.3)
ASC‐H	5 (3.1)
LSIL	26 (16.0)
HSIL	45 (27.8)
SCC	0 (0)
Others	0 (0)
Histology	82 (100)
CIN1	20 (24.4)
CIN2	24 (29.3)
CIN3	35 (42.7)
No dysplasia	3 (3.7)

Abbreviations: ASC‐H, atypical squamous cells, not excluding a high‐grade squamous intraepithelial lesion; ASC‐US, atypical squamous cells of undetermined significance; CIN, cervical intraepithelial neoplasia; HSIL, high‐grade squamous intraepithelial lesion; LSIL, low‐grade squamous intraepithelial lesion; NILM, negative for intraepithelial lesion or malignancy; SCC, squamous cell carcinoma.

Ninety women underwent histological examination. Among them, we excluded eight women whose data were obtained earlier than 1 year preceding the day of informed consent. Histological classifications were as follows: CIN1, 24.4%; CIN2, 29.3%; CIN3, 42.7%; and no CIN, 3.7%.

### Overall, positive, and negative concordance rates between self‐ and physician‐sampling HPV tests

The overall concordance rate between physician‐ and self‐sampling HPV testing was 88.5% (95% confidence interval [CI]: 82.6%–92.9%) (Table 2). The kappa coefficient, which was calculated to evaluate the concordance, was 0.76 (95% CI: 0.66–0.86), indicating a high concordance rate. The *p* value of the McNemar test was 0.36, indicating no significant difference in the test results between physician‐ and self‐sampling. The positive and negative concordance rates between self‐ and physician‐sampling for HPV testing were 88.3% (95% CI: 80.5%–93.8%) and 88.7% (95% CI: 78.1%–95.3%), respectively. These results demonstrated strong agreement between the two sampling methods.

**TABLE 2:** Overall, positive, and negative concordance rates of self‐ and physician‐sampling HPV testing

		HPV self‐sampling	
		Positive	Negative
HPV physician‐sampling	Positive	91	12
	Negative	7	55
Overall concordance rate (95% CI):	88.5% (82.6–92.9)		
Positive concordance rate (95% CI):	88.3% (80.5–93.8)		
Negative concordance rate (95% CI):	88.7% (78.1–95.3)		
Kappa coefficient (95%):	0.76 (0.66–0.86)		
*p* Value for the McNemar test:	0.36		

Abbreviations: CI, confidence interval; HPV, human papillomavirus.

### Distribution of the results of self‐ and physician‐sampling HPV tests based on the HPV genotype

#### Overall, positive, and negative concordance rates according to HPV genotype

Results of self‐ and physician‐sampling according to HPV genotype was shown on Table 3a. For HPV16, the overall concordance rate was 96.4% (95% CI: 92.3%–98.7%), the kappa coefficient was 0.85 (95% CI: 0.74–0.97), and the *p* value for the McNemar test was 0.22 (Table 3b). For HPV18, the overall concordance rate was 98.8% (95% CI: 95.7%–99.9%), the kappa coefficient was 0.74 (95% CI: 0.40–1.00), and the *p* value for the McNemar test was 0.50. For other HPV genotypes, the overall concordance rate was 90.3% (95% CI: 84.7%–94.4%), the kappa coefficient was 0.81 (95% CI: 0.72–0.90), and the *p* value for the McNemar test was 0.08. Tables 3a and 3b indicated that there was no difference between the two sampling methods based on the HPV genotype.

**TABLE 3a:** Results of self‐ and physician‐sampling according to HPV genotype

			HPV self‐sampling	
			Positive	Negative
HPV physician‐sampling	HPV16	Positive	21	1
		Negative	5	138
	HPV18	Positive	3	0
		Negative	2	160
	Others*	Positive	74	12
		Negative	4	75

*Note*: Others*: HPV31, 33, 35, 39, 45, 51, 52, 56, 58, 59, 66, and 68.

Abbreviation: HPV, human papillomavirus.

**TABLE 3b:** Overall concordance rate, positive concordance rate, and negative concordance rate according to HPV genotype

	Overall concordance rate % (95% CI)	Positive concordance rate % (95% CI)	Negative concordance rate % (95% CI)	Kappa coefficient (95% CI)	McNemar test
HPV16	96.4 (92.3–98.7)	95.5 (77.2–99.9)	96.5 (92.0–98.9)	0.85 (0.74–0.97)	0.22
HPV18	98.8 (95.7–99.9)	100 (29.2–100.0)	98.8 (95.6–99.9)	0.74 (0.40–1.00)	0.50
Others*	90.3 (84.7–94.4)	86.0 (76.9–92.6)	94.9 (87.5–98.6)	0.81 (0.72–0.90)	0.08

*Note*: Others*: HPV31, 33, 35, 39, 45, 51, 52, 56, 58, 59, 66, and 68.

Abbreviations: CI, confidence interval; HPV, human papillomavirus.

#### Overall, positive, and negative concordance rates in women with normal or abnormal cytology (other than NILM)

Among the 162 women who underwent cervical cytology, there were 71 and 91 women with normal (NILM) and abnormal cytology (other than NILM) (Tables 4a and 4b), respectively. The positive and negative concordance rates were calculated for each group. There were no women diagnosed with atypical glandular cells, adenocarcinoma in situ, or adenocarcinoma. The HPV‐positive rates between self‐ and physician‐sampling among women with normal cytology were 31.0% and 33.8%, respectively. The HPV‐positive rates between self‐ and physician‐sampling among women with abnormal cytology were 80.2% and 83.5%, respectively. Among women with normal cytology, the proportion of negative self‐collected to negative physician‐collected samples was 93.6% (44/47). Moreover, among the women with abnormal cytology, the proportion of positive self‐collected to positive physician‐collected samples was 90.8% (69/76).

**TABLE 4a:** Overall, positive, and negative concordance rates in women with normal cytology (NILM)

		HPV self‐sampling	
		Positive	Negative
HPV physician‐sampling	Positive	19	5
	Negative	3	44
Overall concordance rate, % (95% CI):	88.7 (79.0–95.0)		
Positive concordance rate, % (95% CI):	79.2 (57.8–92.9)		
Negative concordance rate, % (95% CI):	93.6 (82.5–98.7)		
Kappa coefficient (95% CI):	0.74 (0.58–0.91)		
*p* Value for the binomial test	0.73		

Abbreviations: CI, confidence interval; HPV, human papillomavirus; NILM, negative for intraepithelial lesion or malignancy.

**TABLE 4b:** Overall, positive, and negative concordance rates in women with abnormal cytology (other than NILM)

		HPV self‐sampling	
		Positive	Negative
HPV physician‐sampling	Positive	69	7
	Negative	4	11
Overall concordance rate, % (95% CI):	87.9 (79.4–93.8)		
Positive concordance rate, % (95% CI):	90.8 (81.9–96.2)		
Negative concordance rate, % (95% CI):	73.3 (44.9–92.2)		
Kappa coefficient (95% CI):	0.59 (0.38–0.81)		
*p* Value for the McNemar test:	0.55		

Abbreviations: CI, confidence interval; HPV, human papillomavirus; NILM, negative for intraepithelial lesion or malignancy.

The HPV‐negative and ‐positive concordance rates were high in women with normal and abnormal cytology. Tables 4a and 4b demonstrate that there was no difference between the two sampling methods based on cytology.

### Comparison of positive rates between pathological grades

The HPV‐positive rate was calculated for 82 women with cytology tests performed within 1 year from the day of informed consent (Table 5). For women with CIN1, the HPV‐positive rates were 95.0% (19/20) and 90.0% (18/20) for self‐ and physician‐sampling, respectively. For women with CIN2, the HPV‐positive rates were 75.0% (18/24) and 91.7% (22/24) for self‐ and physician‐sampling, respectively. For women with CIN3, the HPV‐positive rate was 85.7% (30/35) and 88.6% (31/35) for self‐ and physician‐sampling, respectively (Table 5). As shown in Table 5, there were no significant between‐method differences according to the histological grades.

**TABLE 5:** Comparison of positive rates between histological grades

	HPV self‐sampling	HPV physician‐sampling	McNemar test
	Positive rate, % (95% CI)	Positive rate, % (95% CI)	
No dysplasia (*n* = 3)	100.0 (29.2–100.0)	100.0 (29.2–100.0)	–
CIN1 (*n* = 20)	95.0 (75.1–99.9)	90.0 (68.3–98.8)	*p* = 1.00
CIN2 (*n* = 24)	75.0 (53.3–90.2)	91.7 (73.0–99.0)	*p* = 0.13
CIN3 (*n* = 35)	85.7 (69.7–95.2)	88.6 (73.3–96.8)	*p* = 1.00

Abbreviations: CI, confidence interval; CIN, cervical intraepithelial neoplasia; HPV, human papillomavirus.

### Questionnaire results regarding the usability of the HSD‐ST

Most participants (97.6%, 161/165) were using the HSD‐ST for the first time (Table 6). Regarding the usability of the HSD‐ST, 95.8% (158/165) responded that “it was easy,” 95.8% (158/165) responded that “it was not embarrassing,” and 97.6% (161/165) responded that they would use it again in the future. The questionnaire results demonstrated that the participants had an extremely good response to the HSD‐ST.

**TABLE 6:** Results of questionnaire about the HPV sponge‐type self‐sampling device (HSD‐ST)

Is this your first time to use a self‐sampling device for HPV testing?		
Yes	No	No answer
161 (97.6%)	2 (1.2%)	2 (1.2%)
How was your experience using it?		
It was easy.	It was difficult.	No answer
158 (95.8%)	7 (4.2%)	0 (0%)
How was the usability?		
It was not embarrassing.	It was embarrassing.	No answer
158 (95.8%)	6 (3.6%)	1 (0.6%)
Do you want to use the self‐sampling device for HPV testing again in the future?		
I want to use.	I do not want to use.	No answer
161 (97.6%)	3 (1.8%)	1 (0.6%)

Abbreviation: HPV, human papillomavirus.

## DISCUSSION

In Japan, the participation rate in the cervical cancer screening program is low.^3^ In some countries, self‐sampling HPV testing is implemented in national programs to improve participation rates,^9^, ^10^ which is expected to be the case in Japan.

There has been increasing interest in self‐sampling HPV testing worldwide given the decreasing participation rates due to the COVID‐19 pandemic.^11^ However, several challenges remain. Among them, there is no clearly recommended self‐sampling device.

We evaluated the feasibility of the HSD‐ST, which has a sponge section for insertion (Figure 1), since we hypothesized that it would involve less pain and abnormal genital bleeding compared with the brush‐type HPV self‐sampling device (HSD‐BT). Moreover, we postulated that the HSD‐ST (Home Smear Set Plus®, Asica Medical Industry Co., Ltd.), which was developed and made in Japan, would be the most suitable device for Japanese women.

In our study, the overall concordance rate between self‐ and physician‐sampling was 88.5%, with the McNemar test revealing no significant between‐method difference (*p* = 0.36). The calculated kappa coefficient was 0.76, indicating good consistency. Previous studies reported that the calculated kappa coefficient for HSD‐ST and HSD‐BT was 0.7–0.8.^12^, ^13^, ^14^, ^15^ The concordance rate between self‐ and physician‐sampling HPV testing was extremely high.

No studies have directly compared HSD‐ST and HSD‐BT, which are widely used for self‐sampling HPV testing.^16^ The calculated kappa coefficients of HSD‐BT ranged from 0.7 to 0.8. Moreover, another study using a similar HSD‐ST (Home Smear Set Plus®) and HPV testing (Cobas 4800 system HPV) reported an overall concordance rate of 96.3%.^17^ Moreover, the overall concordance rate between the HSD‐ST and HSD‐BT was similar.

In Japan, five studies have examined self‐sampling HPV testing, with two and three studies on HSD‐ST and HSD‐BT, respectively,^17^, ^18^, ^19^, ^20^ which revealed a high concordance rate between HSD‐ST and HSD‐BT.

We examined whether HPV16 and HPV18, which are the most likely to progress to cancer,^17^ were missed by HSD‐ST. The concordance rates for the HPV16 and HPV18 genotypes were 96.4% and 98.8%, respectively. In HSD‐BT, the concordance rates of the HPV16 and HPV18 genotypes using our HPV testing method were 99.0% and 100%, respectively.^13^ There were similar concordance rates between HSD‐ST and HSD‐BT. Similarly, we observed that the HSD‐ST could reliably detect HPV16 and HPV18 genotypes.

To implement HSD‐ST in cervical cancer screening programs, it is important to determine its positive rate (sensitivity) for abnormal cytology. In our study, there was no difference in the positive rate (sensitivity) between HSD‐ST and physician‐sampling (80.2% [73/91] and 83.5% [76/91], respectively). The corresponding values in a previous study were 51.4% (19/37) and 56.8% (21/37), respectively,^17^ with no statistically significant between‐group differences.

It is important to determine the positive rate for CIN2 or worse using the HSD‐ST to determine its feasibility in cervical cancer screening programs. In our study, the sensitivity rates for CIN2 or worse were 81.4% (48/59) and 89.8% in self‐ and physician‐sampling, respectively, exhibiting no between‐method difference in sensitivity. In 2018, Arbyn et al. conducted an updated meta‐analysis on 56 accuracy studies and 25 participation trials, regardless of the device.^16^ The sensitivity rates for CIN2 or worse were 75% and 88% in self‐ and physician‐sampling, respectively, with no significant between‐method difference in sensitivity for PCR‐based HPV testing.^16^ In 2020, Onuma et al. reported that the sensitivity for CIN2 or worse was 100% for both HSD‐BT and physician‐sampling.^18^ Our findings suggest that HSD‐ST could be comparable to HSD‐BT and be implemented into cervical cancer screening programs.

The limitation of this study is not to directly compare that the feasibility of HSD‐ST and HSD‐BT were not directly compared, but the performance of HSD‐ST was evaluated based on the results of physician‐sampling. This study serves as a first step toward the introduction of HSD‐ST into the cancer screening program in Japan. The next research step is confirming that HSD‐ST is non‐inferior to HSD‐BT, which is the main self‐sampling device, regarding the cancer detection rate among women visiting gynecologic clinics. The final step will be directly comparing the cancer detection rate between HSD‐ST and HSD‐BT in cervical cancer screening programs.

In conclusion, this is the first study to show the positive rate (sensitivity) for CIN2 or worse using HSD‐ST. We observed an extremely high concordance rate and comparable positive rate (sensitivity) for CIN2 or worse between HPV self‐ and physician‐sampling testing. Our findings demonstrate the feasibility and usability of the HSD‐ST. Introducing the HSD‐ST in cervical cancer screening programs could improve the participation rate. A future study comparing HSD‐ST and HSD‐BT in the cervical cancer screening program is required.

## Author Contributions

None.

## Conflict of Interest

The authors declare no conflicts of interest other than those noted in acknowledgments or funding information.

## Data Availability

The data that support the findings of this study are available on request from the corresponding author. The data are not publicly available due to privacy or ethical restrictions.
